# Piperazine-1,4-diium bis­(2-carb­oxy-1*H*-pyrazole-4-carboxyl­ate) tetra­hydrate

**DOI:** 10.1107/S160053681003655X

**Published:** 2010-09-18

**Authors:** Xin-Hui Zhou

**Affiliations:** a Key Laboratory for Organic Electronics & Information Displays (KLOEID) and Institute of Advanced Materials (IAM), Nanjing University of Posts & Telecommunications (NUPT), Nanjing 210046, People’s Republic of China

## Abstract

The asymmetric unit of the title compound, C_4_H_12_N_2_^2+^·2C_5_H_3_N_2_O_4_^−^·4H_2_O, comprises one-half of a piperazine-1,4-diium cation, which lies on an inversion centre, a 2-carb­oxy-1*H*-pyrazole-4-carboxyl­ate anion and two water mol­ecules. An extensive network of inter­molecular O—H⋯O, N—H⋯O, N—H⋯N and C—H⋯O hydrogen bonds between the cations, anions and water mol­ecules leads to a three-dimensional supra­molecular framework.

## Related literature

For 3,5-pyrazole­dicarb­oxy­lic acid, see: King *et al.* (2003 ▶); Pan *et al.* (2001 ▶). For reference structural data, see: Li & Su (2007 ▶); Reviriego *et al.* (2006 ▶).
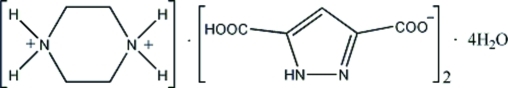


## Experimental

### 

#### Crystal data

C_4_H_12_N_2_·2C_5_H_3_N_2_O_4_·4H_2_O
*M* *_r_* = 470.41
Monoclinic, 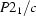

*a* = 8.3363 (13) Å
*b* = 16.246 (3) Å
*c* = 7.3930 (11) Å
β = 90.812 (3)°
*V* = 1001.2 (3) Å^3^
*Z* = 2
Mo *K*α radiation
μ = 0.14 mm^−1^
*T* = 291 K
0.15 × 0.14 × 0.12 mm

#### Data collection

Bruker SMART APEX CCD diffractometer
Absorption correction: multi-scan (*SADABS*; Bruker, 2000 ▶) *T*_min_ = 0.980, *T*_max_ = 0.984
5265 measured reflections
1946 independent reflections
1524 reflections with *I* > 2σ(*I*)
*R*_int_ = 0.051

#### Refinement

*R*[*F* ^2^ > 2σ(*F* ^2^)] = 0.059
*wR*(*F* ^2^) = 0.147
*S* = 1.10
1946 reflections
169 parameters
H atoms treated by a mixture of independent and constrained refinement
Δρ_max_ = 0.41 e Å^−3^
Δρ_min_ = −0.23 e Å^−3^

### 

Data collection: *SMART* (Bruker, 2000 ▶); cell refinement: *SAINT* (Bruker, 2000 ▶); data reduction: *SAINT*; program(s) used to solve structure: *SHELXTL* (Sheldrick, 2008 ▶); program(s) used to refine structure: *SHELXTL*; molecular graphics: *DIAMOND* (Brandenburg, 2008 ▶); software used to prepare material for publication: *SHELXTL*.

## Supplementary Material

Crystal structure: contains datablocks I, global. DOI: 10.1107/S160053681003655X/om2361sup1.cif

Structure factors: contains datablocks I. DOI: 10.1107/S160053681003655X/om2361Isup2.hkl

Additional supplementary materials:  crystallographic information; 3D view; checkCIF report

## Figures and Tables

**Table 1:** Hydrogen-bond geometry (Å, °)

*D*—H⋯*A*	*D*—H	H⋯*A*	*D*⋯*A*	*D*—H⋯*A*
N1—H1⋯O5^i^	0.84 (3)	1.93 (3)	2.746 (3)	167 (3)
O2—H2⋯O4^ii^	0.87 (3)	1.65 (3)	2.520 (2)	175 (3)
N3—H3*A*⋯O4	0.88 (3)	2.36 (3)	2.918 (3)	121 (2)
N3—H3*A*⋯N2	0.88 (3)	2.01 (3)	2.865 (3)	162 (3)
N3—H3*B*⋯O3^iii^	0.88 (3)	2.20 (3)	2.999 (3)	150 (3)
O5—H5*B*⋯O6	0.85 (4)	2.00 (4)	2.831 (3)	166 (3)
O5—H5*A*⋯O6^iv^	0.92 (4)	1.96 (4)	2.833 (3)	157 (3)
O6—H6*C*⋯O3^v^	0.93 (3)	1.85 (3)	2.779 (3)	173 (3)
O6—H6*D*⋯O3^vi^	0.76 (3)	2.14 (3)	2.858 (3)	158 (4)
C6—H6*B*⋯O5^vii^	0.97	2.53	3.348 (4)	142
C7—H7*A*⋯O1^viii^	0.97	2.53	3.091 (3)	117

Symmetry codes: (i) 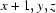; (ii) 
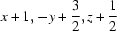
; (iii) 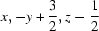; (iv) 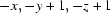; (v) 
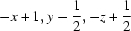
; (vi) 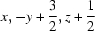; (vii) 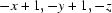; (viii) 
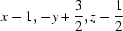
.

**Fig. 1.**
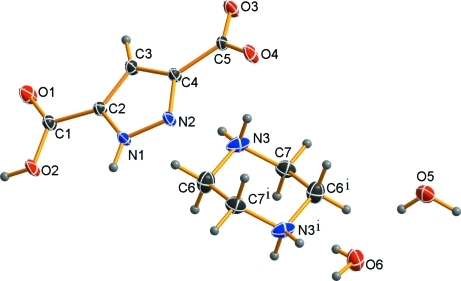
The molecular structure of the title compound with 30% probability displacement ellipsoids. H atoms are shown as small spheres of arbitary radii. [Symmetry code; (i) 1 - x, 1 - y, -z.]

**Fig. 2.**
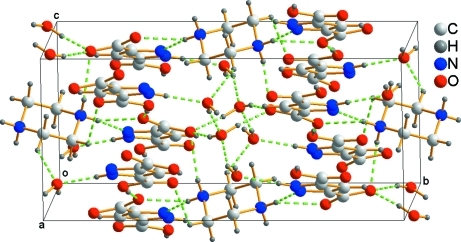
A view of the crystal packing. Hydrogen bonds are indicated by green dashed lines.

## supplementary crystallographic information

### Comment

Hydrogen bonding, as the strongest and most directional intermolecular force, has been intensively investigated in organic crystalline solids. The ligand, 3,5-pyrazoledicarboxylic acid, known both as a multiple proton donor and acceptor, has six potential hydrogen-bond sites involving both the nitrogen atoms of the pyrazole ring and all of the carboxylate O atoms. and it can form mono-, di- and trianionic ligand species through deprotonation (King *et al.* 2003; Pan *et al.* 2001).

We report here the synthesis and structure of piperazine-1,4-diium bis(2-carboxy-1*H*-pyrazole-4-carboxylate) tetrahydrate, as shown in Fig.1, which was obtained from a solution of 3,5-pyrazoledicarboxylic acid, Cd(NO_3_)_2_.4H_2_O and piperazine. Bond distances and angles are normal (Li & Su, 2007; Reviriego *et al.* 2006). The asymmetric unit of the title compound comprises one half of the piperazine-1,4-diium cation, which lies about an inversion centre, a 2-carboxy-1*H*-pyrazole-4-carboxylate anion and two water molecules. In the crystal structure molecules are interlinked by hydrogen bonds (Table 1 and Fig. 2). The 2-carboxy-1*H*-pyrazole-4-carboxylate anoins are interconnected with each other through the O2—H2···O4^iii^ hydrogen bonds. The 2-carboxy-1*H*-pyrazole-4-carboxylate anions are connected with the piperazine-1,4-diium cations through the N3—H3A···O4, N3—H3A···N2, N3—H3B···O3^iv^ and C7—H7A···O1^viii^ hydrogen bonds to form the three-dimensional supramolecular framework.

### Experimental

A mixture of 3,5-pyrazoledicarboxylic acid (0.2 mmol, 34.8 mg), Cd(NO_3_)_2_.4H_2_O (0.1 mmol, 30.8 mg), piperazine (0.2 mmol, 17.2 mg) and H_2_O (8 ml) was sealed in a 15 ml Teflon-lined bomb and heated at 150°C for 5 days. The reaction mixture was slowly cooled to room temperature to obtain the colorless block crystals of (I) suitable for X-ray diffraction analysis.

### Refinement

Hydrogen atoms bonded to the carbon atoms were placed in calculated positions and refined as riding mode, with C—H = 0.93 Å for aromatic H atom, 0.97 Å for methylene H atoms, respectively, and *U*_iso_(H) = 1.2*U*_eq_(C). The H atoms on the O and N atoms were located in difference Fourier map with their bond lengths freely refined and *U*_iso_(H) = 1.2*U*_eq_(O or N).

### Crystal data

**Table:**

C_4_H_12_N_2_·2C_5_H_3_N_2_O_4_·4H_2_O	*F*(000) = 496
*M**_r_* = 470.41	*D*_x_ = 1.560 Mg m^−^^3^
Monoclinic, *P*2_1_/*c*	Mo *K*α radiation, λ = 0.71073 Å
Hall symbol: -P 2ybc	Cell parameters from 1373 reflections
*a* = 8.3363 (13) Å	θ = 2.7–24.0°
*b* = 16.246 (3) Å	µ = 0.14 mm^−^^1^
*c* = 7.3930 (11) Å	*T* = 291 K
β = 90.812 (3)°	Block, white
*V* = 1001.2 (3) Å^3^	0.15 × 0.14 × 0.12 mm
*Z* = 2	

### Data collection

**Table:**

Bruker SMART APEX CCD diffractometer	1946 independent reflections
Radiation source: fine-focus sealed tube	1524 reflections with *I* > 2σ(*I*)
graphite	*R*_int_ = 0.051
phi and ω scans	θ_max_ = 26.0°, θ_min_ = 2.4°
Absorption correction: multi-scan (*SADABS*; Bruker, 2000)	*h* = −10→10
*T*_min_ = 0.980, *T*_max_ = 0.984	*k* = −13→19
5265 measured reflections	*l* = −8→9

### Refinement

**Table:**

Refinement on *F*^2^	Primary atom site location: structure-invariant direct methods
Least-squares matrix: full	Secondary atom site location: difference Fourier map
*R*[*F*^2^ > 2σ(*F*^2^)] = 0.059	Hydrogen site location: inferred from neighbouring sites
*wR*(*F*^2^) = 0.147	H atoms treated by a mixture of independent and constrained refinement
*S* = 1.10	*w* = 1/[σ^2^(*F*_o_^2^) + (0.0729*P*)^2^] where *P* = (*F*_o_^2^ + 2*F*_c_^2^)/3
1946 reflections	(Δ/σ)_max_ < 0.001
169 parameters	Δρ_max_ = 0.41 e Å^−^^3^
0 restraints	Δρ_min_ = −0.23 e Å^−^^3^

### Special details

**Table:**

Geometry. All e.s.d.'s (except the e.s.d. in the dihedral angle between two l.s. planes) are estimated using the full covariance matrix. The cell e.s.d.'s are taken into account individually in the estimation of e.s.d.'s in distances, angles and torsion angles; correlations between e.s.d.'s in cell parameters are only used when they are defined by crystal symmetry. An approximate (isotropic) treatment of cell e.s.d.'s is used for estimating e.s.d.'s involving l.s. planes.
Refinement. Refinement of *F*^2^ against ALL reflections. The weighted *R*-factor *wR* and goodness of fit *S* are based on *F*^2^, conventional *R*-factors *R* are based on *F*, with *F* set to zero for negative *F*^2^. The threshold expression of *F*^2^ > σ(*F*^2^) is used only for calculating *R*-factors(gt) *etc*. and is not relevant to the choice of reflections for refinement. *R*-factors based on *F*^2^ are statistically about twice as large as those based on *F*, and *R*- factors based on ALL data will be even larger.

### Fractional atomic coordinates and isotropic or equivalent isotropic displacement parameters (Å^2^)

**Table:**

	*x*	*y*	*z*	*U*_iso_*/*U*_eq_	
C1	1.1506 (3)	0.78226 (15)	0.2962 (3)	0.0267 (6)	
C2	0.9861 (3)	0.77149 (14)	0.2216 (3)	0.0233 (6)	
C3	0.8727 (3)	0.82885 (15)	0.1741 (3)	0.0256 (6)	
H3	0.8827	0.8858	0.1785	0.031*	
C4	0.7391 (3)	0.78344 (14)	0.1178 (3)	0.0226 (5)	
C5	0.5785 (3)	0.81182 (15)	0.0528 (3)	0.0246 (6)	
C6	0.6653 (4)	0.51108 (17)	−0.0379 (5)	0.0455 (8)	
H6A	0.7149	0.4999	0.0790	0.055*	
H6B	0.7496	0.5242	−0.1223	0.055*	
C7	0.4237 (3)	0.56416 (16)	0.1024 (4)	0.0380 (7)	
H7A	0.3509	0.6107	0.1070	0.046*	
H7B	0.4666	0.5548	0.2233	0.046*	
N1	0.9196 (2)	0.69726 (13)	0.1933 (3)	0.0266 (5)	
H1	0.962 (3)	0.6522 (18)	0.220 (4)	0.032*	
N2	0.7696 (2)	0.70254 (12)	0.1296 (3)	0.0262 (5)	
N3	0.5556 (3)	0.58226 (14)	−0.0224 (3)	0.0399 (7)	
H3A	0.604 (4)	0.6268 (19)	0.020 (4)	0.048*	
H3B	0.514 (4)	0.5944 (18)	−0.130 (4)	0.048*	
O1	1.2097 (2)	0.85001 (11)	0.3144 (3)	0.0454 (6)	
O2	1.2193 (2)	0.71272 (11)	0.3390 (3)	0.0360 (5)	
H2	1.311 (4)	0.7215 (17)	0.394 (4)	0.043*	
O3	0.5431 (2)	0.88609 (10)	0.0779 (3)	0.0360 (5)	
O4	0.4901 (2)	0.75870 (11)	−0.0188 (3)	0.0358 (5)	
O5	0.0107 (3)	0.53912 (14)	0.2765 (4)	0.0528 (7)	
H5A	−0.077 (5)	0.510 (2)	0.316 (5)	0.063*	
H5B	0.093 (5)	0.539 (2)	0.346 (5)	0.063*	
O6	0.2621 (2)	0.51492 (13)	0.5325 (3)	0.0476 (6)	
H6C	0.323 (4)	0.472 (2)	0.486 (5)	0.057*	
H6D	0.320 (4)	0.549 (2)	0.556 (5)	0.057*	

### Atomic displacement parameters (Å^2^)

**Table:**

	*U*^11^	*U*^22^	*U*^33^	*U*^12^	*U*^13^	*U*^23^
C1	0.0215 (13)	0.0285 (14)	0.0299 (15)	0.0002 (11)	−0.0059 (11)	0.0006 (11)
C2	0.0199 (12)	0.0226 (13)	0.0272 (14)	−0.0006 (10)	−0.0039 (10)	0.0002 (10)
C3	0.0205 (13)	0.0193 (12)	0.0369 (15)	−0.0020 (9)	−0.0056 (10)	−0.0016 (10)
C4	0.0189 (12)	0.0203 (12)	0.0286 (14)	0.0011 (10)	−0.0045 (10)	−0.0004 (10)
C5	0.0159 (12)	0.0228 (13)	0.0351 (15)	−0.0025 (10)	−0.0040 (10)	0.0030 (11)
C6	0.0399 (17)	0.0443 (18)	0.052 (2)	−0.0078 (14)	0.0024 (14)	−0.0050 (15)
C7	0.0455 (17)	0.0286 (15)	0.0398 (17)	0.0027 (12)	−0.0021 (14)	−0.0035 (12)
N1	0.0191 (11)	0.0186 (11)	0.0417 (14)	0.0025 (9)	−0.0092 (9)	0.0022 (9)
N2	0.0169 (10)	0.0215 (11)	0.0398 (13)	−0.0001 (8)	−0.0103 (9)	0.0011 (9)
N3	0.0616 (17)	0.0210 (12)	0.0370 (15)	−0.0144 (11)	−0.0069 (12)	−0.0007 (10)
O1	0.0326 (11)	0.0284 (11)	0.0745 (16)	−0.0083 (8)	−0.0248 (10)	0.0010 (10)
O2	0.0195 (9)	0.0284 (10)	0.0597 (14)	−0.0004 (8)	−0.0183 (9)	0.0030 (9)
O3	0.0239 (10)	0.0218 (10)	0.0622 (14)	0.0044 (8)	−0.0093 (9)	−0.0002 (9)
O4	0.0221 (9)	0.0282 (10)	0.0566 (13)	−0.0002 (8)	−0.0167 (9)	−0.0048 (9)
O5	0.0395 (12)	0.0361 (12)	0.0826 (19)	0.0042 (10)	−0.0040 (12)	0.0144 (11)
O6	0.0345 (12)	0.0334 (12)	0.0748 (17)	−0.0035 (9)	−0.0015 (11)	−0.0107 (11)

### Geometric parameters (Å, °)

**Table:**

C1—O1	1.213 (3)	C6—H6B	0.9700
C1—O2	1.304 (3)	C7—N3	1.476 (4)
C1—C2	1.481 (3)	C7—C6^i^	1.504 (4)
C2—N1	1.342 (3)	C7—H7A	0.9700
C2—C3	1.369 (3)	C7—H7B	0.9700
C3—C4	1.395 (3)	N1—N2	1.333 (3)
C3—H3	0.9300	N1—H1	0.84 (3)
C4—N2	1.341 (3)	N3—H3A	0.88 (3)
C4—C5	1.489 (3)	N3—H3B	0.88 (3)
C5—O4	1.248 (3)	O2—H2	0.87 (3)
C5—O3	1.256 (3)	O5—H5A	0.92 (4)
C6—N3	1.480 (4)	O5—H5B	0.85 (4)
C6—C7^i^	1.504 (4)	O6—H6C	0.93 (3)
C6—H6A	0.9700	O6—H6D	0.76 (3)
			
O1—C1—O2	125.7 (2)	H6A—C6—H6B	108.0
O1—C1—C2	121.4 (2)	N3—C7—C6^i^	109.4 (2)
O2—C1—C2	112.9 (2)	N3—C7—H7A	109.8
N1—C2—C3	106.9 (2)	C6^i^—C7—H7A	109.8
N1—C2—C1	122.8 (2)	N3—C7—H7B	109.8
C3—C2—C1	130.3 (2)	C6^i^—C7—H7B	109.8
C2—C3—C4	105.2 (2)	H7A—C7—H7B	108.2
C2—C3—H3	127.4	N2—N1—C2	112.3 (2)
C4—C3—H3	127.4	N2—N1—H1	122.4 (18)
N2—C4—C3	110.4 (2)	C2—N1—H1	125.1 (19)
N2—C4—C5	119.6 (2)	N1—N2—C4	105.21 (19)
C3—C4—C5	130.0 (2)	C7—N3—C6	111.1 (2)
O4—C5—O3	126.1 (2)	C7—N3—H3A	106 (2)
O4—C5—C4	116.5 (2)	C6—N3—H3A	113 (2)
O3—C5—C4	117.5 (2)	C7—N3—H3B	108 (2)
N3—C6—C7^i^	111.0 (2)	C6—N3—H3B	110 (2)
N3—C6—H6A	109.4	H3A—N3—H3B	108 (3)
C7^i^—C6—H6A	109.4	C1—O2—H2	110.5 (18)
N3—C6—H6B	109.4	H5A—O5—H5B	117 (3)
C7^i^—C6—H6B	109.4	H6C—O6—H6D	107 (3)

Symmetry codes: (i) −*x*+1, −*y*+1, −*z*.

### Hydrogen-bond geometry (Å, °)

**Table:**

*D*—H···*A*	*D*—H	H···*A*	*D*···*A*	*D*—H···*A*
N1—H1···O5^ii^	0.84 (3)	1.93 (3)	2.746 (3)	167 (3)
O2—H2···O4^iii^	0.87 (3)	1.65 (3)	2.520 (2)	175 (3)
N3—H3A···O4	0.88 (3)	2.36 (3)	2.918 (3)	121 (2)
N3—H3A···N2	0.88 (3)	2.01 (3)	2.865 (3)	162 (3)
N3—H3B···O3^iv^	0.88 (3)	2.20 (3)	2.999 (3)	150 (3)
O5—H5B···O6	0.85 (4)	2.00 (4)	2.831 (3)	166 (3)
O5—H5A···O6^v^	0.92 (4)	1.96 (4)	2.833 (3)	157 (3)
O6—H6C···O3^vi^	0.93 (3)	1.85 (3)	2.779 (3)	173 (3)
O6—H6D···O3^vii^	0.76 (3)	2.14 (3)	2.858 (3)	158 (4)
C6—H6B···O5^i^	0.97	2.53	3.348 (4)	142
C7—H7A···O1^viii^	0.97	2.53	3.091 (3)	117

Symmetry codes: (ii) *x*+1, *y*, *z*; (iii) *x*+1, −*y*+3/2, *z*+1/2; (iv) *x*, −*y*+3/2, *z*−1/2; (v) −*x*, −*y*+1, −*z*+1; (vi) −*x*+1, *y*−1/2, −*z*+1/2; (vii) *x*, −*y*+3/2, *z*+1/2; (i) −*x*+1, −*y*+1, −*z*; (viii) *x*−1, −*y*+3/2, *z*−1/2.
